# Toward a Clinically Actionable, Electronic Health Record–Based Machine Learning Model to Forecast 90-Day Change in Hemoglobin A1c in Youth With Type 1 Diabetes: Feasibility and Model Development Study

**DOI:** 10.2196/69142

**Published:** 2025-09-25

**Authors:** Erin M Tallon, David D Williams, Cintya Schweisberger, Colin Mullaney, Brent Lockee, Diana Ferro, Craig A Vandervelden, Mitchell S Barnes, Angelica Cristello Sarteau, Anna R Kahkoska, Susana R Patton, Sanjeev Mehta, Ryan McDonough, Marcus Lind, Leonard D'Avolio, Mark A Clements

**Affiliations:** 1 Division of Pediatric Endocrinology and Diabetes, Children's Mercy Kansas City, 2401 Gillham Road, Kansas City, MO, United States, 1 8166014023; 2 Department of Pediatrics, UMKC School of Medicine, Kansas City, MO, United States; 3 Institute for Data Science and Informatics, University of Missouri-Columbia, Columbia, MO, United States; 4 Division of Health Services and Outcomes Research, Children's Mercy Kansas City, Kansas City, MO, United States; 5 Blue Circle Health, Boston, MA, United States; 6 Preventive and Predictive Medicine, IRCCS, Bambino Gesù Children's Hospital, Rome, Italy; 7 Department of Nutrition, University of North Carolina at Chapel Hill, Chapel Hill, NC, United States; 8 Center for Healthcare Delivery Science, Nemours Children's Health, Jacksonville, FL, United States; 9 Joslin Diabetes Center, Boston, MA, United States; 10 Department of Pediatrics, Arkansas Children's Northwest, Springdale, AR, United States; 11 Department of Medicine, NU-Hospital Group, Uddevalla, Sweden; 12 Department of Molecular and Clinical Medicine, University of Gothenburg, Gothenburg, Sweden; 13 Department of Medicine, Sahlgrenska University Hospital, Gothenburg, Sweden; 14 Harvard Medical School, Boston, MA, United States; 15 Mass General Brigham, Boston, MA, United States

**Keywords:** adolescent, AI, artificial intelligence, clinical decision support, EHR, electronic health records, glycemic control, HbA1c, hemoglobin A1c, machine learning, pediatric, population health, prediction, real-world data, T1D, type 1 diabetes, youth

## Abstract

**Background:**

Clinicians currently lack an effective means for identifying youth with type 1 diabetes (T1D) who are at risk for experiencing glycemic deterioration between diabetes clinic visits. As a result, their ability to identify youth who may optimally benefit from targeted interventions designed to address rising glycemic levels is limited. Although electronic health records (EHR)–based risk predictions have been used to forecast health outcomes in T1D, no study has investigated the potential for using EHR data to identify youth with T1D who will experience a clinically significant rise in glycated hemoglobin (HbA_1c_) ≥0.3% (approximately 3 mmol/mol) between diabetes clinic visits.

**Objective:**

We aimed to evaluate the feasibility of using routinely collected EHR data to develop a machine learning model to predict 90-day unit-change in HbA_1c_ (in % units) in youth (aged 9‐18 y) with T1D. We assessed our model’s ability to augment clinical decision-making by identifying a percent change cut point that optimized identification of youth who would experience a clinically significant rise in HbA_1c_.

**Methods:**

From a cohort of 2757 youth with T1D who received care from a network of pediatric diabetes clinics in the Midwestern United States (January 2012-August 2017), we identified 1743 youth with 9643 HbA_1c_ observation windows (ie, 2 HbA_1c_ measurements separated by 70‐110 d, approximating the 90-day time interval between routine diabetes clinic visits). We used up to 5 years of youths’ longitudinal EHR data to transform 17,466 features (demographics, laboratory results, vital signs, anthropometric measures, medications, diagnosis codes, procedure codes, and free-text data) for model training. We performed 3-fold cross-validation to train random forest regression models to predict 90-day unit-change in HbA_1c_(%).

**Results:**

Across all 3 folds of our cross-validation model, the average root-mean-square error was 0.88 (95% CI 0.85‐0.90). Predicted HbA_1c_(%) strongly correlated with true HbA_1c_(%) (*r*=0.79; 95% CI 0.78‐0.80). The top 10 features impacting model predictions included postal code, various metrics related to HbA_1c_, and the frequency of a diagnosis code indicating difficulty with treatment engagement. At a clinically significant percent rise threshold of ≥0.3% (approximately 3 mmol/mol), our model’s positive predictive value was 60.3%, indicating a 1.5-fold enrichment (relative to the observed frequency that youth experienced this outcome [3928/9643, 40.7%]). Model sensitivity and positive predictive value improved when thresholds for clinical significance included smaller changes in HbA_1c_, whereas specificity and negative predictive value improved when thresholds required larger changes in HbA_1c_.

**Conclusions:**

Routinely collected EHR data can be used to create an ML model for predicting unit-change in HbA_1c_ between diabetes clinic visits among youth with T1D. Future work will focus on optimizing model performance and validating the model in additional cohorts and in other diabetes clinics.

## Introduction

### Background

Type 1 diabetes (T1D), an immune-mediated chronic disease that affects more than 1 in 300 youth in the United States, is characterized by significant to near-total loss of endogenous insulin production [1,2]. Given insulin’s critical role in maintaining glucose homeostasis, the most immediate and pervasive downstream effect of insulin deficiency is persistent, life-threatening hyperglycemia that must be identified through frequent glucose monitoring and managed with lifelong administration of exogenous insulin [1].

Youth with T1D attend routine (often quarterly) diabetes clinic visits where clinicians use glycated hemoglobin (HbA_1c_) testing to assess glycemic status [3,4]. Considered the gold standard for monitoring long-term glycemia in diabetes, HbA_1c_ testing provides an objective measure of an individual’s mean blood glucose during the previous 2‐3 months [3,5]. To achieve glycemic goals, youth with T1D are increasingly being encouraged to adopt sophisticated diabetes technologies, such as hybrid closed-loop insulin pumps and continuous glucose monitoring (CGM) systems [6,7]. Concurrent with the rising availability of these technologies and a strong research base linking HbA_1c_ with the development of diabetes complications, the American Diabetes Association has incrementally lowered its recommended HbA_1c_ goals for youth with diabetes [4,8].

Despite increased adoption of advanced diabetes technologies over time, data from the T1D Exchange indicated that between 2010‐2012 and 2016‐2018, mean HbA_1c_ in US youth with T1D rose from 7.8% (62 mmol/mol) to 8.4% (68 mmol/mol); and in 2016‐2018, only 16% (686/4346) of youth were meeting the American Diabetes Association’s (then) recommended HbA_1c_ goal of <7.5% (<58 mmol/mol) [6]. A separate analysis of 2015‐2016 data indicated that fewer than 20% (1817/9685) of US youth with T1D less than the age of 18 years had an HbA_1c_<7.5% (58 mmol/mol); and fewer than 10% (690/9685) of youth had an HbA_1c_<7% (53 mmol/mol) [9]. Previous research has shown that 1 in 5 youth with T1D experience an increasing HbA_1c_ trajectory between the ages of 8 and 18 years [10].

Through a phenomenon known as “metabolic memory,” periods of hyperglycemia are known to increase risk for diabetes-related microvascular and macrovascular complications for >10 years following initial exposure [11]. A similar—but beneficial—legacy effect is observed in individuals with T1D who are exposed to near-normal glycemia and later experience more favorable long-term diabetes outcomes, even when glycemic levels later rise [11,12]. These findings point to a critical need to optimize the early identification of youth who are candidates for targeted interventions to improve deteriorating glycemia.

The increasing availability of real-world clinical data housed in electronic health records (EHR) is generating opportunities to investigate population-level health outcomes, develop classification and risk prediction models to augment clinical decision-making, and accelerate diagnostic and therapeutic discovery [13-15]. Machine learning (ML) has been used to meaningfully advance understanding of numerous clinical outcomes in individuals with diabetes [16-18], and EHR-based risk predictions have been leveraged to generate insights across the health-disease spectrum, including T1D [19-21].

Given the multifactorial etiology of rising glycemic levels in youth with T1D, it remains difficult to identify youth who are at the highest risk of experiencing increased HbA_1c_ between routine diabetes clinic visits. To date, no study has investigated the feasibility of or potential for using EHR data to develop a predictive model to identify youth with T1D who will experience a clinically significant rise in HbA_1c_ between clinic visits. Such a model could augment clinical decision-making and facilitate initiation of interventions that increase behaviors known to improve glycemia in high-risk youth.

### Objective

We sought to evaluate the feasibility of using ML to identify youth (aged 9‐18 y) with T1D who were candidates for behavioral and care delivery interventions designed to reduce or prevent a predicted rise in HbA_1c_. To do so, we used routinely collected EHR data to develop an interpretable and clinically actionable ML model to forecast unit-change (ie, increase or decrease, in % units) in HbA_1c_ in 90 days. We then evaluated the ability of our model to augment clinical decision-making by identifying a percent-change cut point that optimized identification of youth who experienced a clinically significant rise in HbA_1c_ at their subsequent diabetes clinic encounter.

## Methods

### Study Design

We applied the random forest (RF) regression algorithm to longitudinal EHR data to develop a model to forecast 90-day unit-change in HbA_1c_ (in % units). We used RF due to its utility for constructing accurate, noise-resilient ML models from high-dimensional data [22,23]. To evaluate our model’s ability to identify youth who, based on predicted rise in HbA_1c_, were true candidates for intervention, we evaluated the sensitivity, specificity, positive predictive value (PPV), and negative predictive value (NPV) of predicted versus actual change in HbA_1c_ at several cut points: ≥0.3%, ≥0.4%, ≥0.5%, and ≥0.6% (approximately 3 mmol/mol, 4 mmol/mol, 5 mmol/mol, and 7 mmol/mol, respectively).

### Source Data and Study Cohort

Using data extracted from Oracle Health EHR (formerly Cerner Millenium Electronic Medical Record System; Nashville, Tennessee) [24], we used diagnosis code and laboratory data to identify a cohort of 2757 youth with T1D who received care from a network of pediatric diabetes clinics in the Midwestern United States between January 2012 and August 2017. Criteria used to identify this T1D cohort are provided in Multimedia Appendix 1.

### HbA_1c_ Measurements and Observation Windows

For youth with T1D, we identified health encounters that were associated with HbA_1c_ measurements (ie, laboratory and point-of-care HbA_1c_ measurements) and HbA_1c_ observation windows that met inclusion criteria. Each HbA_1c_ observation window comprised 2 documented HbA_1c_ measurements (from a single individual) separated by a time interval of 70‐110 days. The 70‐ to 110-day time interval was selected to approximate the 3-month (ie, 90-day) time interval between regularly scheduled diabetes clinic visits.

Certain encounters with HbA_1c_ data were excluded from consideration and therefore not included in any HbA_1c_ observation windows. HbA_1c_ values documented at or shortly after T1D diagnosis tend to be more extreme than those documented at subsequent time points (ie, after an individual with T1D begins receiving regular insulin injections) [25,26]. As such, each youth’s first-documented encounter with an HbA_1c_ value was excluded under the assumption that a youth’s first HbA_1c_ measurement may have been obtained at the time of T1D diagnosis. We also excluded data from encounters where youth were <9 years old, as the incidence of clinically significant rise in HbA_1c_ is less common in this age group [6,27].

We excluded observation windows associated with HbA_1c_ measurements that were separated by <70 days or >110 days, as well as those where the first encounter for a given HbA_1c_ observation window (ie, the index encounter) was associated with an HbA_1c_ of >12% (>108 mmol/mol). The latter exclusion criterion was used because individuals with an HbA_1c_ of >12% (>108 mmol/mol) were already considered ideal candidates for intervention. Encounter-level data from all HbA_1c_ observation windows that met inclusion criteria were included in our final dataset, which could include data from multiple HbA_1c_ observation windows per individual.

### Outcome Definition

The forecasted outcome was unit-change in HbA_1c_ (in % units) at the end of 90 days. After predicting each youth’s percent change in HbA_1c_ in 90 days (ie, at the time of the follow-up encounter), we used various thresholds to determine an HbA_1c_ percent rise cut point that optimized identification of individuals who were true candidates for intervention at the time of their index encounter: ≥0.3%, ≥0.4%, ≥0.5%, and ≥0.6% (approximately 3 mmol/mol, 4 mmol/mol, 5 mmol/mol, and 7 mmol/mol, respectively). We considered these cut points to be clinically relevant and actionable, given that a long-term decrease of ≥0.3% (3 mmol/mol) in HbA_1c_ is associated with reduced risk of long-term diabetes complications [28].

### Data Extraction

We used SQL queries to comprehensively extract up to approximately 5 years (January 2012-August 2017) of structured and unstructured EHR data for each youth with index and follow-up encounter data for at least 1 qualifying HbA_1c_ observation window. These data included demographics, laboratory results, vital signs, anthropometric measures, encounter locations, medications, diagnosis codes, procedure codes, structured clinical vocabulary codes, and free-text data from diabetes- and non–diabetes-related clinical notes, messages, and reports.

Demographic data included sex (female, male), age, ethnicity (non-Hispanic, Hispanic), race (White, Black or African American, Asian, American Indian or Alaska Native, Native Hawaiian or Pacific Islander, and other), primary language (eg, English or Spanish), health plan type; and postal code (3- and 4-digit postal code prefixes). Additional extracted data included up to approximately 5 years of all available laboratory test results, clinical event and observation data, vital signs (heart rate, respiratory rate, oxygen saturation, and blood pressure), anthropometric measures (weight, height, and BMI), and medications (mapped to standard generic drug names [29]). We also extracted diagnosis codes (ie, *ICD-9* [*International Classification of Diseases, Ninth Revision*], ICD-10 [*International Statistical Classification of Diseases, Tenth Revision*], and Systematized Nomenclature of Medicine Clinical Terms [SNOMED CT] codes), procedure codes (ie, Current Procedural Terminology [CPT] codes); and other structured clinical vocabulary codes (ie, SNOMED CT).

We chose not to include data generated by diabetes devices (eg, automated insulin delivery and CGM systems). Early on, we observed that HbA_1c_ was easiest to predict in youth who used diabetes devices that generate diabetes data (eg, glucose levels) in real time. However, since most diabetes centers do not have broad or ready access to device data in near-real time, we sought to evaluate the potential of using only EHR data to predict HbA_1c_.

### Feature Engineering

We engineered features using data documented during all available historical encounters, as well as during HbA_1c_ observation window index and follow-up encounters. Processes used to transform variables into features for ML varied by data type. In all, our feature engineering processes generated 17,466 input features for model fitting.

### Numeric Variables

For numeric variables (eg, laboratory results, weight, and vital signs), we created features by calculating summary metrics (ie, mean, slope, and SD). In general, we created 2 sets of features for each numeric variable, based on proximity of the measurements to the HbA_1c_ observation window’s index encounter. One set of features was created using data documented during the 12 months preceding (and at) the index encounter. A second set was created using all available EHR data documented before (and at) the index encounter. For example, we created 2 features for mean HbA_1c_: one calculated using the previous 12 months of HbA_1c_ data (up to and including the index encounter), and the other calculated using all available HbA_1c_ data (up to and including the index encounter). Given the intrinsic insensitivity of RF to numerical outliers, we did not alter or drop outliers from the data. Once all numerical features were created, missing numerical values were imputed using the population median.

Each youth’s diagnostic (ie, first) HbA_1c_ result was included as a separate feature, as was the HbA_1c_ result documented at the observation window’s index encounter. Because research suggests that youth with T1D can be grouped into one of several HbA_1c_ trajectory clusters [10], we created an HbA_1c_ trajectory feature by using k-means clustering [30] to assign youth to 1 of 4 clusters based on their quarterly HbA_1c_ measurements.

### Categorical Variables

We used data documented at the observation window’s index encounter to create features from demographic data (eg, age, race, ethnicity, primary language, health plan type, and postal code). For each categorical demographic variable, we used the StringIndexer feature transformer to convert the categories associated with each variable into numeric indices, thus creating a single feature for each of these variables [31].

We used Clinical Classification Software Revised (CCSR), developed by the Agency for Healthcare Research and Quality, to group *ICD-10* codes into meaningful categories [32]. Thereafter, each CCSR category and each *ICD-9*, *ICD-10*, SNOMED CT, and CPT code was treated as a separate variable. We created 2 sets of features for each of these separate variables, based on how many times each had been assigned to the individual relative to the observation window’s index encounter. One set of features was created by calculating the frequency that each had been assigned to the individual during the 12 months preceding (and at) the index encounter. The second set was created using all available EHR data documented before (and at) the index encounter. Absence of diagnosis, procedure, or structured clinical vocabulary codes was presumed to reflect true absence, rather than missingness, of these data variables.

Medication variables were similarly transformed into 2 sets of features based on how often each medication had been prescribed relative to the index encounter. One set of features was created by calculating the frequency that each medication had been prescribed to the individual during the 12 months preceding (and at) the index encounter. The second set was created using all available medication data documented before (and at) the index encounter. Encounter frequencies were similarly calculated and included as separate features. Absence of medication and encounter data was presumed to reflect true absence of these data.

### Natural Language Processing

We used term frequency–inverse document frequency (TF-IDF) vectorization, a natural language processing technique, to process free-text data from clinical notes, messages, and reports. In TF-IDF vectorization, words (ie, tokens) are first converted into a matrix of token counts [33]. The matrix is then transformed into a normalized TF-IDF representation that most heavily weights tokens that occur infrequently across the entire corpus of available text [33]. As such, TF-IDF is used to assign the highest weight to words that have the most discriminating power. After ranking by weight, we constrained the total number of features generated via TF-IDF vectorization to 250 single-word terms and 250 two-word terms, each of which had to be present in at least 5 documents.

### Model Development and Evaluation

RF uses bootstrap aggregation and random feature sampling to independently train a series of uncorrelated decision tree regressors, known as “weak learners” [22,23,34-36]. Predictions from this ensemble of weak learners are averaged to produce a single “strong learner” with improved prediction accuracy [23]. Relative to many other ML methods, the RF algorithm presents several key advantages, including decreased risk of overfitting, straightforward calculation of the degree to which individual input features contribute to model predictions, and robustness to missing data [22,34].

After randomly splitting the entire dataset into 3 nonoverlapping data subsets, we used 3-fold cross-validation to recursively fit RF regressors to 2 of the 3 subsets and then evaluate model performance on the third, held-out subset. We used 3-fold (rather than 5- or 10-fold) cross-validation due to the large number of HbA_1c_ observation windows included in our analysis, as well as our desire to reduce variance in the estimated performance of our model. Hyperparameters used for model fitting are presented in Table 1. Model performance was evaluated by averaging the mean absolute error (MAE) and the root-mean-square error (RMSE)—the SD of the residuals [37]—across all 3 cross-validation models.

**Table 1.** Hyperparameter values used for random forest regressor model training. A complete list of hyperparameter keys accepted by the random forest regressor algorithm and definitions of each can be found on the web [38]. Hyperparameters not listed below were set to default values.

Hyperparameter	Value used	Default value
NumTrees	40	20
MaxDepth	7	5
MaxBins	128	32
MinInstancesPerNode	8	1
FeatureSubsetStrategy	“onethird”	“onethird”
Impurity	“variance”	“variance”
MinInfoGain	0.0	0.0
MinWeightFractionPerNode	0.0	0.0
SubsamplingRate	1.0	1.0

Decision tree regressors are grown by recursively splitting on features to maximize impurity reduction [39]. Feature splits that reduce impurity by maximally reducing variance are considered important; thus, the features that are split to maximize reduction in variance are also deemed important [39,40]. We evaluated feature importance by calculating and ranking the mean reduction in variance associated with only those features that were used by all 3 of our cross-validation models to forecast HbA_1c_.

We used Python (version 3) and Scala (version 2; Programming Methods Laboratory at École Polytechnique Fédérale de Lausanne) to clean and transform the data. ML analyses were conducted using the Apache Spark MLlib (version 2) ML library [41].

### Statistical Analysis

Pearson *r* correlations were used to assess the strength and direction of the relationship between actual and predicted HbA_1c_ values. We also used sensitivity, specificity, PPV, and NPV as clinical performance metrics to aid in identifying a predicted HbA_1c_ percent rise threshold that would facilitate optimal capture of youth who would experience a clinically significant rise in HbA_1c_ in 90 days.

Summary statistics, correlations, RMSE, MAE, and sensitivity, specificity, PPV, and NPV metrics were assessed using Stata/SE (Stata standard edition) software (version 18.5; StataCorp) [42].

### Ethical Considerations

Clinical and model output data were collected and coded in an institutional review board–approved research data repository at Children’s Mercy Kansas City (Kansas City, Missouri; IRB #11120355) that met the requirements for a waiver of written informed consent as outlined in 45 CFR 46.116.

## Results

### Overview

**Figure 1.**
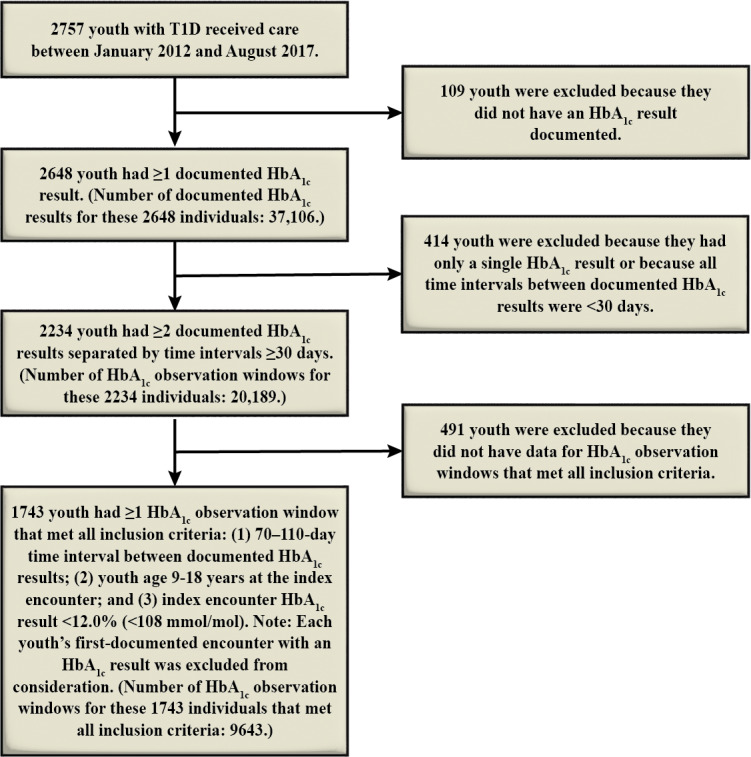
Flowchart depicting inclusion and exclusion criteria for the study cohort and for glycated hemoglobin observation windows. Abbreviations: HbA_1c_: glycated hemoglobin; T1D: type 1 diabetes.

Out of 2757 youth with T1D, 1743 youth (63.2%) had one or more HbA_1c_ observation windows (n=9643) that met inclusion criteria (Figure 1).

Characteristics of the entire cohort that met inclusion criteria are summarized in Table 2. The observed frequencies that youth experienced a rise in HbA_1c_ that exceeded each percent change cut points (≥0.3%, ≥0.4%, ≥0.5%, and ≥0.6% [approximately 3 mmol/mol, 4 mmol/mol, 5 mmol/mol, 7 mmol/mol]) were 40.7%, 35.6%, 30.8%, and 26.5%, respectively. Characteristics of observations included in each nonoverlapping K-fold are summarized in Table 3.

**Table 2.** Demographic and clinical characteristics of 1743 youth with glycated hemoglobin observation windows that met inclusion criteria.

Demographic and clinical characteristics	All HbA_1c_^a^ observation windows (n=9643)	Index encounter of each youth’s first HbA_1c_ observation window (n=1743)
Age (y), mean (SD)	13.8 (2.6)	12.9 (2.7)
Sex, n (%)		
Female	4599 (47.7)	844 (48.4)
Male	5044 (52.3)	899 (51.6)
Unknown	0 (0)	0 (0)
Race, n (%)		
White	8196 (85)	1449 (83.1)
Black or African American	616 (6.4)	133 (7.6)
Asian	53 (0.5)	12 (0.7)
American Indian or Alaska Native	42 (0.4)	8 (0.5)
Native Hawaiian or Pacific Islander	8 (0.1)	3 (0.2)
Other	63 (0.7)	10 (0.6)
Unknown	665 (6.9)	128 (7.3)
Ethnicity, n (%)		
Non-Hispanic or non-Latino	8978 (93.1)	1620 (93.0)
Hispanic or Latino	656 (6.8)	121 (6.9)
Unknown	9 (0.1)	2 (0.1)
HbA_1c_ at index encounter (%), mean (SD)	8.6 (1.3)	8.5 (1.5)
HbA_1c_ at index encounter (mmol/mol), mean (SD)	70 (14.2)	69 (16.4)
Change in HbA_1c_^b^ (%), median (IQR)	0.1 (–0.4 to 0.6)	0.1 (–0.4 to 0.7)
Change in HbA_1c_^b^ (mmol/mol), median (IQR)	1 (–4 to 7)	1 (–4 to 8)
HbA_1c_ increase, n (%)		
≥0.3%	3928 (40.7)	763 (43.8)
≥0.4%	3435 (35.6)	662 (38)
≥0.5%	2966 (30.8)	580 (33.3)
≥0.6%	2552 (26.5)	498 (28.6)

a HbA_1c_: glycated hemoglobin.

b Change in HbA_1c_: (HbA_1c_ at the observation window’s follow-up encounter)–(HbA_1c_ at the observation window’s index encounter).

**Table 3.** Demographic and clinical characteristics of youth with glycated hemoglobin observation windows included in each K-fold subset.

Demographic and clinical characteristics	HbA_1c_^a^ observation windows: fold 1 (n=3151)	HbA_1c_ observation windows: fold 2 (n=3129)	HbA_1c_ observation windows: fold 3 (n=3363)
Youth, n (%)	1291 (41.0)	1288 (41.2)	1381 (41.1)
Age (y), mean (SD)	13.9 (2.6)	13.8 (2.6)	13.8 (2.6)
Sex, n (%)			
Female	1534 (48.7)	1488 (47.6)	1577 (46.9)
Male	1617 (51.3)	1641 (52.4)	1786 (53.1)
Unknown	0 (0)	0 (0)	0 (0)
Race, n (%)			
White	2690 (85.4)	2658 (85.0)	2848 (84.7)
Black or African American	174 (5.6)	206 (6.6)	236 (7.0)
Asian	14 (0.4)	16 (0.5)	23 (0.7)
American Indian or Alaska Native	17 (0.5)	13 (0.4)	12 (0.4)
Native Hawaiian or Pacific Islander	3 (0.1)	3 (0.1)	2 (0.1)
Other	17 (0.5)	21 (0.6)	25 (0.7)
Unknown	236 (7.5)	212 (6.8)	217 (6.4)
Ethnicity, n (%)			
Non-Hispanic or non-Latino	2911 (92.4)	2928 (93.6)	3139 (93.3)
Hispanic or Latino	236 (7.5)	197 (6.3)	223 (6.6)
Unknown	4 (0.1)	4 (0.1)	1 (0.1)
HbA_1c_^a^ at index encounter (%), mean (SD)	8.6 (1.3)	8.6 (1.3)	8.6 (1.3)
HbA_1c_ at index encounter (mmol/mol), mean (SD)	70 (14)	70 (14)	70 (14)
HbA_1c_ increase ≥0.3%, n (%)	1255 (39.8)	1293 (41.3)	1380 (41)

a HbA_1c_: glycated hemoglobin.

### Model Performance

**Figure 2.**
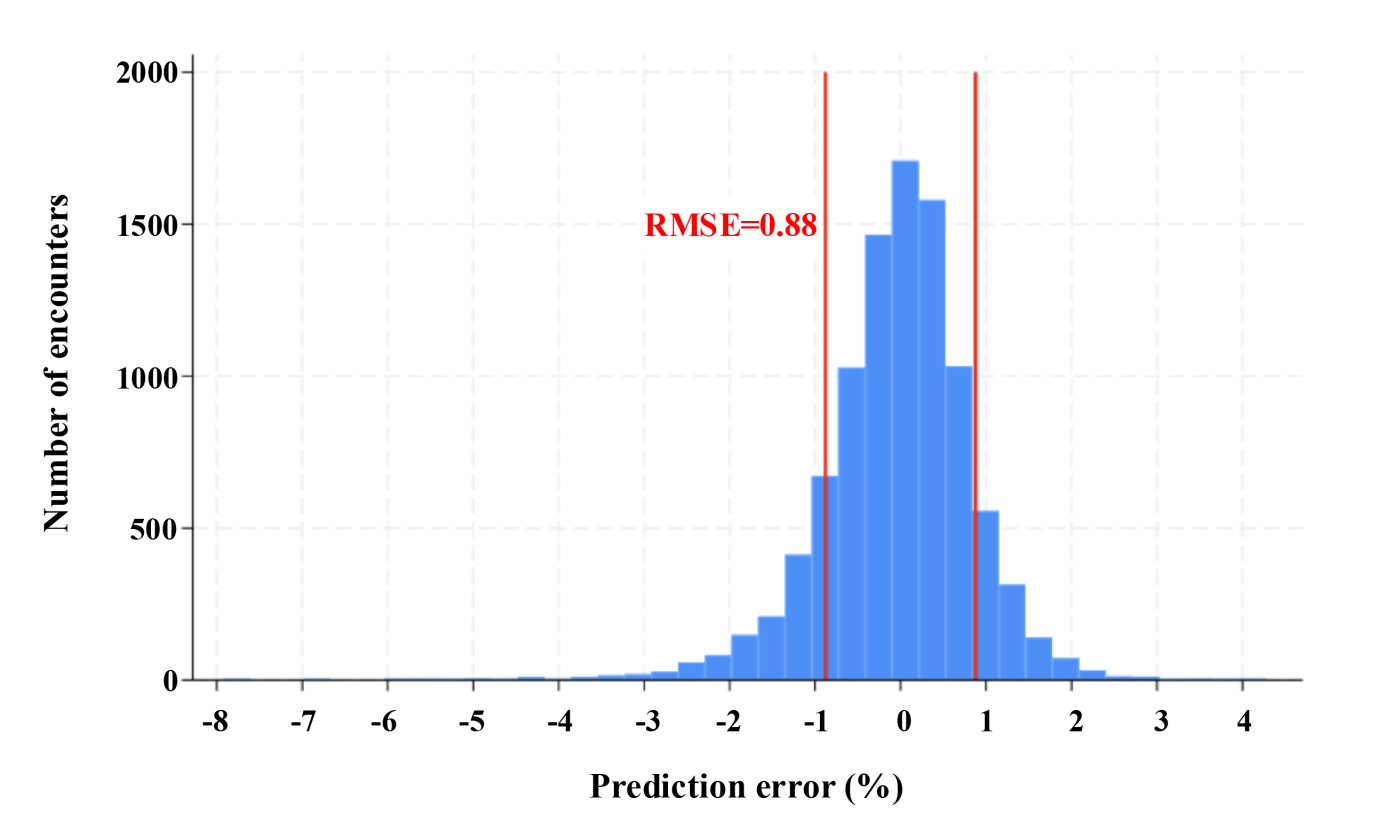
Distribution of the prediction error (ie, residuals) across all 3 cross-validation K-folds. Root-mean-square error is equal to the SD of the prediction error. RMSE: root-mean-square error.

Across all 3 folds of our cross-validation model, average RMSE was 0.88 (Figure 2). Thus, in 68% (6557/9643) of cases (representing one SD), our predictions were within ±0.88% (95% CI 0.85‐0.90) of the true percent change in HbA_1c_. The average MAE across all 3 folds was 0.64 (95% CI 0.63‐0.65). Predicted HbA_1c_(%) strongly correlated with true HbA_1c_(%; *r*=0.79; 95% CI 0.78‐0.80).

### Feature Importance

**Figure 3.**
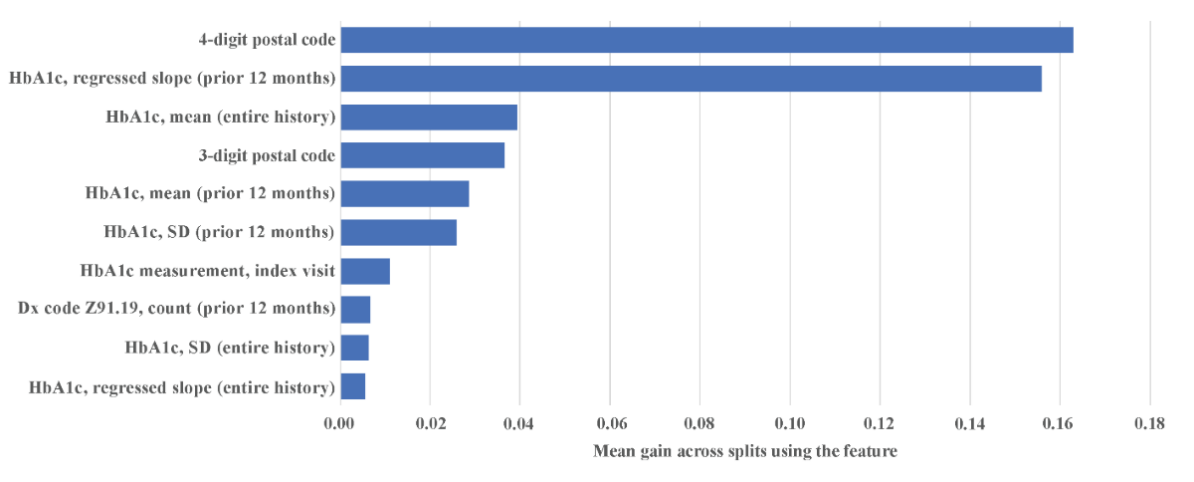
Top 10 most important features for predicting 90-day percent change in glycated hemoglobin, assessed via gain-based feature importance. In random forest regression, gain is a feature importance measure that reflects, for a given feature, the mean increase in node purity (ie, mean reduction in variance) that the feature contributes across all splits in which it is used. Z91.19 is a diagnosis code from the *ICD-10* (*International Classification of Diseases, Tenth Revision*), that is used to code for nonadherence to, or noncompliance with, medical treatment. Dx: diagnosis; HbA_1c_: hemoglobin A_1c_.

Across all 3 folds of our cross-validation model, the top 10 features identified as having the greatest impact on model predictions included postal code, various metrics related to HbA_1c_, and the number of times that the individual had been assigned a diagnosis code indicating difficulty with treatment engagement (Figure 3). The top 30 most important features used to predict percent change in HbA_1c_ are in Multimedia Appendix 2.

### Percent Change Cut Points

Our cross-validation model’s ability to accurately predict change in HbA_1c_ at various percent change cut points is illustrated in Table 4. At each percent change cut point (≥0.3%, ≥0.4%, ≥0.5%, and ≥0.6% [approximately 3 mmol/mol, 4 mmol/mol, 5 mmol/mol, 7 mmol/mol]), PPV was 60.3%, 56.4%, 52.7%, and 53.1%, respectively, indicating an approximately 1.5- to 2-fold enrichment (relative to the observed frequency of each outcome [Table 1]) for identifying youth who would experience a clinically significant rise in HbA_1c_. Sensitivity and PPV improved when predictions involved smaller changes in HbA_1c_, whereas specificity and NPV improved when predictions involved larger changes in HbA_1c_. Sensitivity, specificity, PPV, and NPV metrics for each K-fold are in Multimedia Appendix 3.

**Table 4.** Sensitivity, specificity, positive predictive value, and negative predictive value of predicted versus true percent change in HbA_1c_ across all 3 cross-validation K-folds.

Model metrics at each percent change cut point	Estimate, % (95% CI)
Predicted HbA_1c_^a^ % change: ≥0.3%	
Sensitivity (True HbA_1c_% change: ≥0.3%)	28.7 (27.3-30.2)
Specificity (True HbA_1c_% change: ≥0.3%)	87 (86.1-87.9)
PPV^b^ (True HbA_1c_% change: ≥0.3%)	60.3 (58.1-62.5)
NPV^c^ (True HbA_1c_% change: ≥0.3%)	64 (62.9-65)
Predicted HbA_1c_% change: ≥0.4%	
Sensitivity (True HbA_1c_% change: ≥0.4%)	17.4 (16.1-18.7)
Specificity (True HbA_1c_% change: ≥0.4%)	92.6 (91.9-93.2)
PPV (True HbA_1c_% change: ≥0.4%)	56.4 (53.3-59.4)
NPV (True HbA_1c_% change: ≥0.4%)	66.9 (65.9-67.9)
Predicted HbA_1c_% change: ≥0.5%	
Sensitivity (True HbA_1c_% change: ≥0.5%)	10 (8.9-11.1)
Specificity (True HbA_1c_% change: ≥0.5%)	96 (95.5-96.5)
PPV (True HbA_1c_% change: ≥0.5%)	52.7 (48.4-56.9)
NPV (True HbA_1c_% change: ≥0.5%)	70.6 (69.6-71.5)
Predicted HbA_1c_% change: ≥0.6%	
Sensitivity (True HbA_1c_% change: ≥0.6%)	6.1 (5.2-7.1)
Specificity (True HbA_1c_% change: ≥0.6%)	98.1 (97.7-98.4)
PPV (True HbA_1c_% change: ≥0.6%)	53.1 (47.2-58.9)
NPV (True HbA1% change: ≥0.6%)	74.4 (73.5-75.3)

a HbA_1c_: glycated hemoglobin.

b PPV: positive predictive value (it is the probability that the cases predicted to experience clinically significant rise in HbA_1c_ [at or above each percent rise threshold] did experience that outcome).

c NPV: negative predictive value (it is the probability that the cases not predicted to experience clinically significant rise in HbA_1c_ [at or above each percent rise threshold] did not experience that outcome).

## Discussion

### Principal Findings

We used routinely collected EHR data, including both structured and unstructured data, to establish the feasibility of constructing an interpretable ML model for predicting unit-change in HbA_1c_ (in % units) between quarterly diabetes clinic visits among youth (aged 9‐18 y) with T1D. For those predicted to experience a ≥0.3% (approximately 3 mmol/mol) rise in HbA_1c_ during the following 3 months, PPV was 60.3%, indicating a 1.5-fold enrichment (relative to the observed frequency [40.7%] of this outcome) for identifying youth who would experience a clinically significant rise in HbA_1c_. This finding, which suggests that EHR data may be useful for identifying youth who will experience rising glycemic levels, is clinically relevant given that a long-term increase of ≥0.3% (3 mmol/mol) in HbA_1c_ is associated with increased risk for long-term complications of diabetes [28].

Another key finding was that our model’s sensitivity and PPV were higher when the predicted percent rise threshold was lower (eg, ≥0.3% vs ≥0.4%), whereas specificity and NPV were increased at higher predicted percent rise thresholds (eg, ≥0.4% vs ≥0.3%). We hypothesized that using a higher percent rise threshold would decrease the likelihood of false positives (ie, identifying a youth as someone who would experience a corresponding rise in HbA_1c_ when they did not), and the data supported this conclusion. On the other hand, using a lower percent rise threshold reduced the likelihood of missing those who would experience a clinically significant rise in HbA_1c_. If confirmed in future studies, these findings suggest that using the lowest clinically significant threshold may be useful for guiding clinical decision-making and subsequent initiation of interventions designed to mitigate rising glycemic levels.

We also evaluated our model’s ability to augment clinical decision-making by using PPV and NPV to identify a percent-change cut point that optimized identification of youth who experienced a clinically significant rise in HbA_1c_ at their subsequent diabetes clinic encounter. Although PPV and NPV are considered the metrics of choice for clinical decision-making at the level of an individual person, the selection of desirable PPV and NPV values in a particular use case depends on numerous factors. These factors include considerations about short- and long-term burdens and costs related to over- or undertreatment, associated psychological impacts on individuals receiving care, and short- and long-term costs imposed on the health care system (eg, for increased staffing resources) [43]. Therefore, before implementing this model clinically, it would be important to allow clinicians to provide feedback about the most appropriate thresholds for defining clinically significant rise in HbA_1c_, along with associated PPV and NPV values. For this work, we propose using the ≥0.3% cut-point to maximize capture of high-risk youth who are candidates for behavioral and care delivery interventions designed to reduce or prevent predicted rise in HbA_1c_.

The top features impacting our model’s predictions (ie, postal code, numerous metrics pertaining to HbA_1c_, and history of low treatment engagement) have been shown in previous studies to be associated with elevated glycemic levels. Ample evidence suggests associations between geographic location and geographically linked measures of socioeconomic status (eg, area deprivation, social deprivation, and child opportunity indices) and T1D outcomes, including glycemic levels and diabetic ketoacidosis [44-47]. Previous HbA_1c_ measurements have also been shown to significantly impact ML-based predictions of future HbA_1c_, but previous investigations have only examined this in adults with type 2 diabetes (T2D) [48]. Finally, lower treatment engagement has been shown to have a substantial impact on HbA_1c_ in youth with T1D [49,50]. This evidence collectively underscores the critical need for members of the diabetes care team to partner with affected youth and families to identify resources and tailored strategies for optimizing diabetes self-management behaviors.

Given the widespread use of EHRs in clinical care, as well as the growing volume and availability of these data, there exists tremendous potential for using EHR data to identify and personalize care pathways for improving health outcomes in T1D. Previous work has applied ML to EHR data, for example, to predict the onset of T1D in youth [51], as well as diabetic ketoacidosis in both youth and adults with T1D [19,20,52]. Recent research has focused on applying numerous ML classifiers to medical encounter data to predict HbA_1c_ in individuals with T2D [48]. The area under the receiver operating curve for each of the top 5 best-performing classifiers in the aforementioned study was extremely high (>0.95). Of note, however, these model predictions were binary (ie, HbA_1c_ <7% [<53 mmol/mol] vs ≥7% [≥53 mmol/mol]) rather than continuous and were evaluated in a primarily adult Chinese cohort diagnosed with T2D, limiting generalizability to other populations. Our approach is designed to predict unit change in HbA_1c_ and to give clinicians a simple output (ie, HbA_1c_ will or will not increase by ≥0.3%) for interpretation. This study is the first to use EHR data to predict a clinically significant rise in HbA_1c_ in youth with T1D.

Recent efforts have also explored the use of ML classifiers that use 2 weeks of CGM data to forecast 90-day HbA_1c_ in youth with T1D [53]. The first of these studies used a nested, ensemble learning approach to iteratively predict HbA_1c_ in stages: (1) HbA_1c_ ≤7.5% (58 mmol/mol) or >7.5% (stage 1), (2) HbA_1c_ ≤9% (75 mmol/mol) or >9% (stage 2, after stage 1 was complete), and (3) HbA_1c_ ≤12.5% (113 mmol/mol) or >12.5% (stage 3, after stage 2 was complete) [54]. A subsequent study used few-shot learning followed by K-nearest neighbors to classify transformed images of CGM time series data into multiclass HbA_1c_ intervals [55]. Generalizability of these HbA_1c_ prediction efforts is limited, however, by these methods’ dependence on CGM data and by racial disparities in the relationship between CGM metrics and HbA_1c_ [56].

Currently, CGM systems are neither accessible to nor used by all individuals with T1D. Recent data from the T1D Exchange Quality Improvement Collaborative suggest that only 40%‐50% of US youth with T1D currently use CGM systems [57,58]. Reasons for this are multifactorial and can include reluctance to use CGM technologies, financial constraints, lack of insurance coverage, device-related skin complications, CGM alarm fatigue, and sociodemographic and racial or ethnic disparities in access that adversely impact use of diabetes technologies [58-62]. At this time, CGM data also remain notably absent from most EHRs, are distributed across multiple proprietary commercial software, and are difficult for health systems to access. Although efforts to integrate CGM data into the EHR remain ongoing [63,64], large-scale implementation of these efforts will hinge on the development of CGM-related data standards and a data architecture that supports this integration [65].

In contrast, EHR data are routinely collected on every person receiving care from a given health care institution. These data thus provide a rich, longitudinal source of individual- and population-level health data that can be leveraged in near real-time for ML-driven clinical decision support [13,66]. Even so, the potential for integrating EHR-based ML-driven analytics in health care remains largely unrealized. A 2020 systematic review evaluating the number of clinical prediction models that have been embedded into EHRs noted that fewer than 45 such examples have been published [67]. Of note, only 36% (16/45) of model implementations occurred in outpatient settings, and none of the embedded models were specific to individuals affected by diabetes [67]. These findings highlight a critical gap, as well as opportunity, for leveraging real-world EHR data to facilitate real-time risk prediction and improve diabetes-related health outcomes.

### Limitations and Strengths

A strength of this study is its use of longitudinal EHR data to predict 90-day unit-change in HbA_1c_ in a large cohort of youth with T1D. The scale and granularity of these data facilitated the creation of thousands of data features that we simultaneously analyzed as potential predictors for suboptimal glycemic outcomes. Additional strengths of this study include its use of explainable ML methods for evaluating model predictions and our use of a clinician-led, postmodeling decision analysis to enhance clinicians’ understanding and uptake of model predictions. The relevance of our model is underscored by its ability to forecast 90-day change in HbA_1c_ for all youth receiving care through our regional clinic network, and not only for those using CGM systems.

Several limitations also warrant consideration. The data used in this study originated from a regional network of diabetes clinics in the Midwest United States and may not generalize to other geographic locations or health care settings, to future cohorts using rapidly evolving diabetes treatment technologies, or to more racially or socioeconomically diverse cohorts. External validation of the geographic and demographic “transportability” of this and future iterations of our model will hinge on ensuring that data from different clinical settings are collected in similar ways and standardized according to a common data model. Examples of such data standards include the Observational Medical Outcomes Partnership Common Data Model [68] and the T1D Exchange Quality Improvement Collaborative data specification [69]. As well, EHR data are subject to data entry errors and missing data that inadvertently occur as a part of routine clinical care. EHRs are also characterized by data fragmentation and reflect biases in clinical data collection, documentation, and decision-making [13]. Therefore, results from this and all models constructed using EHR data must be interpreted carefully, given both known and unknown biases that impact model predictions.

Model generalizability could be enhanced by using standardized geographic-based features (eg, an area deprivation index or the Child Opportunity Index [45,47]) rather than zip code, as well as by creating a final prediction model that includes only a limited number of the “top-N features identified via cross-validation. Using additional data preprocessing methods (eg, one-hot encoding) when transforming categorical demographic features (eg, race and ethnicity) for ML would facilitate interpretability of model results pertaining to those features. Model performance may improve with additional hyperparameter tuning. This model’s predictive utility could also be compared with that of models constructed using other ML methods, including other explainable AI methods and deep learning models. Finally, for youth who adopt diabetes technologies, such as CGM and automated insulin delivery systems, the inclusion of diabetes device data would likely significantly augment our model’s predictions.

We acknowledge that translation of this work into clinical practice will be accompanied by various logistical and practical challenges. This study was designed as an “initial step” to evaluate the feasibility of using EHR data to predict change in HbA_1c_. As previously described, additional research is needed to address issues related to model refinement, validation (using data from external organizations, as well as future EHR data collected from our network of diabetes centers), and deployment in clinical settings. Future work can, for example, evaluate whether a limited set of standardized features may be useful for developing a more parsimonious model that can be readily disseminated to other institutions. Once deployed, ongoing monitoring of model performance will also be needed.

Furthermore, we acknowledge that refining and successfully incorporating this approach into clinical and decision workflows will hinge on the collection of additional evidence from future studies with even larger and more diverse patient cohorts, as well as buy-in and trust from both clinicians and patients. Although, in this iteration, our modeling approach yielded a nontrivial number of false positives, we note as well that our model’s performance represents a substantial improvement over existing capabilities. Compared, for example, with initiating interventions randomly or initiating interventions at every diabetes clinic visit (to address youths’ rising glycemic levels, which occurred 40.7% of the time in our cohort), our modeling efforts facilitated pre-emptive identification of rising glucose levels three-fifths of the time. The 1.5-fold risk enrichment demonstrated in this work represents a meaningfully improved opportunity for more targeted initiation and delivery of interventions designed to lower youths’ glucose levels.

### Conclusions

Using EHR data to develop an ML-based prediction model to identify youth who will experience a clinically significant rise in HbA_1c_ between diabetes clinic visits is both timely and feasible. Future research should aim to further optimize model performance, as well as evaluate model performance in racially or ethnically, socioeconomically, and geographically diverse cohorts. Future work is also needed to evaluate whether model results vary by duration of diabetes, use of technology (eg, CGM system users vs nonusers), and insulin delivery modality. Findings from this study may help to inform risk stratification and resource allocation efforts and serve as a catalyst for future quality improvement efforts focused on developing and evaluating personalized strategies and supports for optimizing diabetes self-management behaviors.

## Supplementary material

10.2196/69142 Multimedia Appendix 1 Electronic health records–based identification of a cohort of youth with type 1 diabetes.

10.2196/69142 Multimedia Appendix 2 Top 30 most important features for predicting 90-day percent change in glycated hemoglobin in youth with type 1 diabetes.

10.2196/69142 Multimedia Appendix 3 Sensitivity, specificity, positive predictive value, and negative predictive value of predicted versus true percent change in glycated hemoglobin for each cross-validation K-fold.

## Abbreviations

CCSR: Clinical Classification Software Revised
CGM: continuous glucose monitoring
CPT: Current Procedural Terminology
EHR: electronic health record
HbA_1c_: glycated hemoglobin
ICD-10: *International Statistical Classification of Diseases, Tenth Revision*
ICD-9: *International Classification of Diseases, Ninth Revision*
MAE: mean absolute error
ML: machine learning
NPV: negative predictive value
PPV: positive predictive value
RF: random forest
RMSE: root-mean-square error
SNOMED CT: Systematized Nomenclature of Medicine Clinical Terms
T1D: type 1 diabetes
T2D: type 2 diabetes
TF-IDF: term frequency-inverse document frequency
