# Supplementary material for: Toward a Clinically Actionable, Electronic Health Record–Based Machine Learning Model to Forecast 90-Day Change in Hemoglobin A1c in Youth With Type 1 Diabetes: Feasibility and Model Development Study
Source: JMIR Diabetes. 2025 Sep 25;10:e69142. doi: 10.2196/69142 (PMC12463387; doi:10.2196/69142)
Supplement: Multimedia Appendix 1 [file diabetes-v10-e69142-s001.docx]

## Multimedia Appendix 1

*This is a Multimedia Appendix to a full manuscript published in JMIR Diabetes. For full copyright and citation information see* [*https://dx.doi.org/10.2196/69142*](https://nam04.safelinks.protection.outlook.com/?url=http%3A%2F%2Fdx.doi.org%2F10.2196%2F69142&data=05%7C02%7Cetallon%40cmh.edu%7Ce55ba231941d4cbc7ab308ddc9d99114%7Cfcdc7058dd484a8190b6281159ae72e0%7C0%7C0%7C638888658492218678%7CUnknown%7CTWFpbGZsb3d8eyJFbXB0eU1hcGkiOnRydWUsIlYiOiIwLjAuMDAwMCIsIlAiOiJXaW4zMiIsIkFOIjoiTWFpbCIsIldUIjoyfQ%3D%3D%7C0%7C%7C%7C&sdata=5bMtCyAKvvrM243dX9iar6ievP2U9bJX7M3hZI%2BzQd4%3D&reserved=0)

**Electronic health records-based identification of a cohort of youth with T1D**

Individuals were identified as having type 1 diabetes (T1D) if they met criteria for any one of the following three pathways. Our approach for T1D case identification represents a slightly modified version of the Klompas Optimized T1D Algorithm [1], which is based on the Klompas Primary T1D Algorithm [2].

1. **Pathway 1:** Individuals were identified as having T1D if they had a larger number of T1D diagnosis codes than type 2 diabetes (T2D) diagnosis codes and their most recent diabetes diagnosis code was for T1D. T1D diagnosis codes: 250.%1 (ICD-9), 250.%3 (ICD-9), and E10.% (ICD-10). T2D diagnosis codes: 250.%0 (ICD-9), 250.%2 (ICD-9), and E11.% (ICD-10). (Note: The “%” sign is a wildcard.)

Individuals who had a diagnosis code or a laboratory result indicating a concurrent diagnosis of cystic fibrosis were excluded from the T1D cohort. (In these individuals, a diagnosis of T1D occurs secondary to the primary diagnosis of cystic fibrosis.) Cystic fibrosis diagnosis codes included the following: E84.9 (ICD-10), E84.0 (ICD-10), and E08.9 (ICD-10).

1. **Pathway 2:** Individuals were identified as having T1D if, at any time, they had a C-peptide laboratory result that was <0.6 ng/ml.
2. **Pathway 3:** Individuals were identified as having T1D if, at any time, they had a lab result indicating positivity for one or more of the following: ICA, IAA, IA-2A / ICA512, OR ZnT8 [3].

**Abbreviations:**

IA-2A / ICA512, insulinoma-associated protein 2 autoantibody; IAA, insulin autoantibodies; ICA, islet cell antibodies; ICD-9, International Classification of Disease, 9^th^ Revision; ICD-10, International Classification of Disease, 10^th^ Revision; T1D, type 1 diabetes; T2D, type 2 diabetes; ZnT8, zinc transporter 8 antibodies

## References

1. Raebel MA, Schroeder EB, Goodrich G, Paolino AR, Donahoo WT, Fuller C, et al. Validating type 1 and type 2 diabetes in the Mini-Sentinel Distributed Database using the Surveillance, Prevention, and Management of Diabetes Mellitus (SUPREME-DM) DataLink. 2016.

2. Klompas M, Eggleston E, McVetta J, Lazarus R, Li L, Platt R. Automated detection and classification of type 1 versus type 2 diabetes using electronic health record data. Diabetes Care; 2013;36(4):914-921. doi: 10.2337/dc12-0964

3. Katsarou A, Gudbjörnsdottir S, Rawshani A, Dabelea D, Bonifacio E, Anderson BJ, et al. Type 1 diabetes mellitus. Nat Rev Dis Primers; 2017;3:17016. doi: 10.1038/nrdp.2017.16
