# Supplementary material for: Toward a Clinically Actionable, Electronic Health Record–Based Machine Learning Model to Forecast 90-Day Change in Hemoglobin A1c in Youth With Type 1 Diabetes: Feasibility and Model Development Study
Source: JMIR Diabetes. 2025 Sep 25;10:e69142. doi: 10.2196/69142 (PMC12463387; doi:10.2196/69142)
Supplement: Multimedia Appendix 2 [file diabetes-v10-e69142-s002.docx]

## Multimedia Appendix 2

*This is a Multimedia Appendix to a full manuscript published in JMIR Diabetes. For full copyright and citation information see* [*https://dx.doi.org/10.2196/69142*](https://nam04.safelinks.protection.outlook.com/?url=http%3A%2F%2Fdx.doi.org%2F10.2196%2F69142&data=05%7C02%7Cetallon%40cmh.edu%7Ce55ba231941d4cbc7ab308ddc9d99114%7Cfcdc7058dd484a8190b6281159ae72e0%7C0%7C0%7C638888658492218678%7CUnknown%7CTWFpbGZsb3d8eyJFbXB0eU1hcGkiOnRydWUsIlYiOiIwLjAuMDAwMCIsIlAiOiJXaW4zMiIsIkFOIjoiTWFpbCIsIldUIjoyfQ%3D%3D%7C0%7C%7C%7C&sdata=5bMtCyAKvvrM243dX9iar6ievP2U9bJX7M3hZI%2BzQd4%3D&reserved=0)

**Top 30 most important features for predicting 90-day percent change in HbA_1c_ in youth with type 1 diabetes (T1D).**

Feature importance was assessed via gain-based feature importance. In random forest regression, gain is a feature importance measure that reflects, for a given feature, the mean increase in node purity (i.e., mean reduction in variance) that the feature contributes across all splits in which it is used. Z91.19 is a diagnosis code from the International Classification of Disease, 10^th^ revision, that is used to code for nonadherence to, or noncompliance with, medical treatment.

| **Rank** | **Importance** | **Feature** |
| --- | --- | --- |
| 1 | 0.1629 | 4-digit postal code |
| 2 | 0.1560 | HbA_1c_, regressed slope (prior 12 months) |
| 3 | 0.0393 | HbA_1c_, mean (entire history) |
| 4 | 0.0365 | 3-digit postal code |
| 5 | 0.0286 | HbA_1c_, mean (prior 12 months) |
| 6 | 0.0259 | HbA_1c_, SD (prior 12 months) |
| 7 | 0.0111 | HbA_1c_ measurement, index visit |
| 8 | 0.0067 | Dx code Z91.19, count (prior 12 months) |
| 9 | 0.0064 | HbA_1c_, SD (entire history) |
| 10 | 0.0055 | HbA_1c_, regressed slope (entire history) |
| 11 | 0.0039 | Thyroid stimulating hormone in mcIU/mL, mean (prior 12 months) |
| 12 | 0.0032 | Diastolic BP in mmHg, measured via BP cuff, SD (prior 12 months) |
| 13 | 0.0030 | BMI in kg/m2, regressed slope (entire history) |
| 14 | 0.0029 | Race |
| **Rank** | **Importance** | **Feature** |
| 15 | 0.0029 | HbA_1c_ trajectory cluster, index visit |
| 16 | 0.0028 | BMI in kg/m2, SD (prior 12 months) |
| 17 | 0.0026 | Weight at admission in kg, regressed slope (prior 12 months) |
| 18 | 0.0026 | Systolic BP in mmHg, measured via BP cuff, regressed slope (prior 12 months) |
| 19 | 0.0026 | Systolic BP in mmHg, measured via BP cuff, SD (prior 12 months) |
| 20 | 0.0025 | HDL Cholesterol in mg/dL, mean (prior 12 months) |
| 21 | 0.0025 | Weight in kg, SD (prior 12 months) |
| 22 | 0.0025 | GAD autoantibodies in units/mL, mean (entire history) |
| 23 | 0.0024 | Weight in kg, regressed slope (entire history) |
| 24 | 0.0024 | Weight in kg, regressed slope (prior 12 months) |
| 25 | 0.0023 | Heart rate in beats per minute, regressed slope (prior 12 months) |
| 26 | 0.0023 | Diastolic BP in mmHg, measured via BP cuff, regressed slope (prior 12 months) |
| 27 | 0.0023 | Heart rate in beats per minute, mean (entire history) |
| 28 | 0.0023 | Systolic BP in mmHg, measured via BP cuff, regressed slope (entire history) |
| 29 | 0.0023 | Height/length in cm, SD (entire history) |
| 30 | 0.0022 | HbA_1c_ measurement, first recorded (per person) |

**Abbreviations:**

BP, blood pressure; Dx, diagnosis; HbA_1c_, hemoglobin A_1c_; HDL, high-density lipoprotein; mcIU: microinternational units; mmHg, millimeters of mercury; SD, standard deviation
