# Supplementary material for: Toward a Clinically Actionable, Electronic Health Record–Based Machine Learning Model to Forecast 90-Day Change in Hemoglobin A1c in Youth With Type 1 Diabetes: Feasibility and Model Development Study
Source: JMIR Diabetes. 2025 Sep 25;10:e69142. doi: 10.2196/69142 (PMC12463387; doi:10.2196/69142)
Supplement: Multimedia Appendix 3 [file diabetes-v10-e69142-s003.docx]

## Multimedia Appendix 3

*This is a Multimedia Appendix to a full manuscript published in JMIR Diabetes. For full copyright and citation information see* [*https://dx.doi.org/10.2196/69142*](https://nam04.safelinks.protection.outlook.com/?url=http%3A%2F%2Fdx.doi.org%2F10.2196%2F69142&data=05%7C02%7Cetallon%40cmh.edu%7Ce55ba231941d4cbc7ab308ddc9d99114%7Cfcdc7058dd484a8190b6281159ae72e0%7C0%7C0%7C638888658492218678%7CUnknown%7CTWFpbGZsb3d8eyJFbXB0eU1hcGkiOnRydWUsIlYiOiIwLjAuMDAwMCIsIlAiOiJXaW4zMiIsIkFOIjoiTWFpbCIsIldUIjoyfQ%3D%3D%7C0%7C%7C%7C&sdata=5bMtCyAKvvrM243dX9iar6ievP2U9bJX7M3hZI%2BzQd4%3D&reserved=0)

**Sensitivity, specificity, PPV, and NPV of predicted versus true percent change in HbA_1c_ for each cross-validation K-fold.**

**Fold 1**

|  | Estimate [95% CI] (%) |
| --- | --- |
| *Predicted HbA_1c_ % change: ≥0.3%* |  |
| Sensitivity (True HbA_1c_% change: ≥0.3%) | 29.9 [27.4, 32.5] |
| Specificity (True HbA_1c_ % change: ≥0.3%) | 86.4 [84.8, 87.9] |
| PPV (True HbA_1c_ % change: ≥0.3%) | 59.2 [55.3, 63.1] |
| NPV (True HbA_1c_ % change: ≥0.3%) | 65.1 [63.2, 66.9] |
| *Predicted HbA_1c_ % change: ≥0.4%* |  |
| Sensitivity (True HbA_1c_ % change: ≥0.4%) | 19.6 [17.3, 22.1] |
| Specificity (True HbA_1c_ % change: ≥0.4%) | 92.8 [91.6, 93.9] |
| PPV (True HbA_1c_ % change: ≥0.4%) | 59.6 [54.4, 64.7] |
| NPV (True HbA_1c_ % change: ≥0.4%) | 68.1 [66.3, 69.8] |
| *Predicted HbA_1c_ % change: ≥0.5%* |  |
| Sensitivity (True HbA_1c_ % change: ≥0.5%) | 11.2 [9.3, 13.4] |
| Specificity (True HbA_1c_ % change: ≥0.5%) | 96.5 [95.6, 97.2] |
| PPV (True HbA_1c_ % change: ≥0.5%) | 58.2 [50.7, 65.4] |
| NPV (True HbA_1c_ % change: ≥0.5%) | 71.4 [69.7, 73.0] |
| *Predicted HbA_1c_ % change: ≥0.6%* |  |
| Sensitivity (True HbA_1c_ % change: ≥0.6%) | 6.6 [5.0, 8.5] |
| Specificity (True HbA_1c_ % change: ≥0.6%) | 98.2 [97.6, 98.7] |
| PPV (True HbA_1c_ % change: ≥0.6%) | 55.8 [45.2, 66.0] |
| NPV (True HbA_1c_ % change: ≥0.6%) | 75.5 [73.9, 77.0] |

**Fold 2**

|  | Estimate [95% CI] (%) |
| --- | --- |
| *Predicted HbA_1c_ % change: ≥0.3%* |  |
| Sensitivity (True HbA_1c_ % change: ≥0.3%) | 27.5 [25.1, 30.1] |
| Specificity (True HbA_1c_ % change: ≥0.3%) | 86.8 [85.2, 88.3] |
| PPV (True HbA_1c_ % change: ≥0.3%) | 59.5 [55.5, 63.5] |
| NPV (True HbA_1c_ % change: ≥0.3%) | 63.0 [61.1, 64.9] |
| *Predicted HbA_1c_ % change: ≥0.4%* |  |
| Sensitivity (True HbA_1c_ % change: ≥0.4%) | 14.7 [12.7, 16.9] |
| Specificity (True HbA_1c_ % change: ≥0.4%) | 92.1 [90.9, 93.3] |
| PPV (True HbA_1c_ % change: ≥0.4%) | 51.9 [46.3, 57.4] |
| NPV (True HbA_1c_ % change: ≥0.4%) | 65.3 [63.5, 67.0] |
| *Predicted HbA_1c_ % change: ≥0.5%* |  |
| Sensitivity (True HbA_1c_ % change: ≥0.5%) | 8.9 [7.2, 10.8] |
| Specificity (True HbA_1c_ % change: ≥0.5%) | 95.9 [95.0, 96.7] |
| PPV (True HbA_1c_ % change: ≥0.5%) | 50.3 [42.6, 57.9] |
| NPV (True HbA_1c_ % change: ≥0.5%) | 69.4 [67.7, 71.1] |
| *Predicted HbA_1c_ % change: ≥0.6%* |  |
| Sensitivity (True HbA_1c_ % change: ≥0.6%) | 5.4 [4.0, 7.1] |
| Specificity (True HbA_1c_ % change: ≥0.6%) | 98.2 [97.6, 98.7] |
| PPV (True HbA_1c_ % change: ≥0.6%) | 52.9 [41.9, 63.7] |
| NPV (True HbA_1c_ % change: ≥0.6%) | 73.4 [71.8, 75.0] |

**Fold 3**

|  | Estimate [95% CI] (%) |
| --- | --- |
| *Predicted HbA_1c_ % change: ≥0.3%* |  |
| Sensitivity (True HbA_1c_ % change: ≥0.3%) | 28.8 [26.5, 31.3] |
| Specificity (True HbA_1c_ % change: ≥0.3%) | 87.7 [86.2, 89.2] |
| PPV (True HbA_1c_ % change: ≥0.3%) | 62.1 [58.2, 65.9] |
| NPV (True HbA_1c_ % change: ≥0.3%) | 63.9 [62.1, 65.7] |
| *Predicted HbA_1c_ % change: ≥0.4%* |  |
| Sensitivity (True HbA_1c_ % change: ≥0.4%) | 17.8 [15.6, 20.1] |
| Specificity (True HbA_1c_ % change: ≥0.4%) | 92.7 [91.6, 93.8] |
| PPV (True HbA_1c_ % change: ≥0.4%) | 57.2 [52.0, 62.3] |
| NPV (True HbA_1c_ % change: ≥0.4%) | 67.4 [65.7, 69.1] |
| *Predicted HbA_1c_ % change: ≥0.5%* |  |
| Sensitivity (True HbA_1c_ % change: ≥0.5%) | 9.9 [8.1, 11.9] |
| Specificity (True HbA_1c_ % change: ≥0.5%) | 95.6 [94.7, 96.4] |
| PPV (True HbA_1c_ % change: ≥0.5%) | 49.8 [42.7, 56.8] |
| NPV (True HbA_1c_ % change: ≥0.5%) | 70.9 [69.3, 72.5] |
| *Predicted HbA_1c_ % change: ≥0.6%* |  |
| Sensitivity (True HbA_1c_ % change: ≥0.6%) | 6.4 [4.9, 8.2] |
| Specificity (True HbA_1c_ % change: ≥0.6%) | 97.8 [97.1, 98.3] |
| PPV (True HbA_1c_ % change: ≥0.6%) | 50.9 [41.3, 60.5] |
| NPV (True HbA_1c_ % change: ≥0.6%) | 74.2 [72.7, 75.7] |

**Abbreviations:**

HbA_1c_, hemoglobin A_1c_; NPV, negative predictive value; PPV, positive predictive value
